# Healthy Minds, Healthy Bodies: Enhancing Physical Health in Serious Mental Illness – BSW (Bath, Swindon and Wiltshire) Physical Health Evaluation

**DOI:** 10.1192/bjo.2025.10481

**Published:** 2025-06-20

**Authors:** Elizabeth Ewins, Ian Burgess, Alison Kinge, Josephine Raffan-Burnett

**Affiliations:** AWP, Bath, United Kingdom

## Abstract

**Aims:** The BSW (Bath, Swindon and Wiltshire) Community Physical Health Evaluation aimed to assess the implementation and outcomes of annual physical health checks for individuals with Serious Mental Illness (SMI) within the BSW region. BSW offer an assertive outreach approach for physical health checks by mental health specific nurses and support workers. The evaluation explored compliance with physical health check processes, follow-up practices for abnormal results, and the role of social deprivation in influencing health check uptake. It sought to identify barriers, assess adherence to policy guidelines, and recommend improvements for managing cardio-metabolic risk.

**Methods:** Data for the evaluation was drawn from the AWP (Avon and Wiltshire Partnership) electronic record system, focusing on cardio-metabolic screening forms and associated documentation. The sample period was for the financial year 2022/2023 with a total sample size of 21 service users (SU); 16 SU who received physical health checks, 4 SU who did not and 5 SU who are deceased. A literature review guided the methodological framework, focusing on studies and policies addressing cardio-metabolic risks in SMI populations. Data analysis examined compliance rates, abnormal result follow-ups, and the impact of socioeconomic factors.

**Results:** Compliance with annual physical health checks improved from 67% to 84%, exceeding the national target of 65%, set by NHSE. The rates of high blood pressure, dyslipidaemia and higher risk alcohol use appear to be lower in BSW than national averages. The rates of smoking, raised glucose, and obesity however are higher than national averages. Despite checks being done, the interventions recorded are low (43%) and care plans were in place for only 52% of the service users audited. Service users from socially deprived areas exhibited lower engagement rates, highlighting inequality in service access. Findings also emphasized the importance of assertive engagement strategies and specialized physical health training for mental health professionals.

**Conclusion:** While progress in compliance rates reflects successful implementation efforts, challenges persist in ensuring comprehensive follow-up care and addressing health inequalities. The evaluation recommends enhanced collaboration between secondary and primary care, improved training for staff, and targeted interventions for socially deprived groups to mitigate cardio-metabolic risks. Future efforts should focus on refining data-sharing processes and promoting integrated care to ensure sustained health outcomes for SMI populations.

